# Increased Echogenicity and Radiodense Foci on Echocardiogram and MicroCT in Murine Myocarditis

**DOI:** 10.1371/journal.pone.0159971

**Published:** 2016-08-03

**Authors:** Angela K. Peter, William H. Bradford, Nancy D. Dalton, Yusu Gu, Chieh-Ju Chao, Kirk L. Peterson, Kirk U. Knowlton

**Affiliations:** 1 Department of Medicine, University of California San Diego, San Diego, California, United States of America; 2 BioFrontiers, University of Colorado, Boulder, Colorado, United States of America; 3 Department of Internal Medicine, Mayo Clinic College of Medicine, Phoenix, Arizona, United States of America; 4 Department of Medicine, John H. Stroger Jr. Hospital of Cook County, Chicago, Illinois, United States of America; 5 Intermountain Heart Institute, Intermountain Medical Center, Salt Lake City, Utah, United States of America; Albert Einstein College of Medicine, UNITED STATES

## Abstract

**Objectives:**

To address the question as to whether echocardiographic and/or microcomputed tomography (microCT) analysis can be utilized to assess the extent of Coxsackie B virus (CVB) induced myocarditis in the absence of left ventricular dysfunction in the mouse.

**Background:**

Viral myocarditis is a significant clinical problem with associated inflammation of the myocardium and myocardial injury. Murine models of myocarditis are commonly used to study the pathophysiology of the disease, but methods for imaging the mouse myocardium have been limited to echocardiographic assessment of ventricular dysfunction and, to a lesser extent, MRI imaging.

**Methods:**

Using a murine model of myocarditis, we used both echocardiography and microCT to assess the extent of myocardial involvement in murine myocarditis using both wild-type mice and CVB cleavage-resistant dystrophin knock-in mice.

**Results:**

Areas of increased echogenicity were only observed in the myocardium of Coxsackie B virus infected mice. These echocardiographic abnormalities correlated with the extent of von Kossa staining (a marker of membrane permeability), inflammation, and fibrosis. Given that calcium phosphate uptake as imaged by von Kossa staining might also be visualized using microCT, we utilized microCT imaging which allowed for high-resolution, 3-dimensional images of radiodensities that likely represent calcium phosphate uptake. As with echocardiography, only mice infected with Coxsackie B virus displayed abnormal accumulation of calcium within individual myocytes indicating increased membrane permeability only upon exposure to virus.

**Conclusions:**

These studies demonstrate new, quantitative, and semi-quantitative imaging approaches for the assessment of myocardial involvement in the setting of viral myocarditis in the commonly utilized mouse model of viral myocarditis.

## Introduction

Inflammation of the heart muscle, or myocarditis, refers most commonly to inflammation caused by exposure to external pathogens such as parasites, bacteria, or viruses but may also be caused by autoimmune disorders [1]. Of the infectious agents, Coxsackie B viruses (CVB), members of the enteroviral genus, are among the most common etiological agents known to cause myocarditis [2, 3]. The clinical presentation of myocarditis is highly variable, including subclinical disease, cardiac arrhythmias, cardiomyopathy, and the rapidly progressive and potentially fatal disease known as fulminant myocarditis [4]. Myocarditis can eventually progress to cardiac dilation and heart failure in both children and adults [3–5].

Mouse models of myocarditis are used extensively to study the pathophysiology of viral myocarditis. In order to assess the severity of myocarditis in mice, histologic analysis is frequently used. While echocardiography can be used to assess changes in ventricular function following CVB infection, abnormalities in function are often detected in only the most severely diseased ventricles and the degree of dysfunction does not always correlate with the severity of myocardial involvement. Recently, there has been considerable progress made in diagnosing myocarditis utilizing cardiac magnetic resonance imaging (MRI) in patients with suspected disease [6]. While MRI is also possible in mice [7, 8], the expense, availability of proper equipment, and individuals with suitable training make routine use of this imaging modality challenging. In addition, other imaging techniques have higher resolution than MRI. Improved imaging to allow assessment of myocardial involvement over time and at high resolution could contribute significantly to our understanding of pathogenesis of myocarditis.

During 2D echocardiography of CVB3 infected mice, abnormal echogenicity was noted within the myocardium of mice infected with CVB3. We hypothesized that the extent of echogenicity would correlate with the pathologic evidence of myocardial involvement from myocarditis. Accordingly, we analyzed three well-characterized pathological markers of myocarditis; inflammation, fibrosis and von Kossa staining. We found that each of these correlated to the extent of myocardial echogenicity observed on echocardiography. Having observed these echocardiographic changes and given that the von Kossa stain is a marker for calcium phosphate uptake, we further sought to determine whether microCT could be utilized similarly to visualize changes in the myocardium of infected mice and found that it allowed for high-resolution, 3-dimensional images of radiodensities that likely represents calcium phosphate uptake in the heart of infected mice.

## Materials and Methods

### Virus

CVB3 was derived from the infectious cDNA copy of the cardiotrophic H3 variant of CVB3. We determined viral titer using the PFU assay in HeLa cells, as described previously [9].

### Generating Mice Expressing Mutant Dystrophin That Cannot Be Cleaved by Protease 2A, Dys^KI^

Knock-in of a dystrophin mutation preventing cleavage by protease 2A was generated as previously described [9]. Mice were genotyped to identify the knock-in construct using a DysKI-S primer (5′-TCTCTAGGAGAGGTCTCTC) and a DysKI-AS primer (5′-ACCCCACAATCTTGCACATG). Dys^KI^ mice were backcrossed for 3 to 11 generations with C3H mice (Charles River Laboratories) for viral infection experiments. Control mice were wild type C3H mice from the same litters, Dys^WT^.

### CVB3 Infection

Male Dys^KI^, mice and their littermate controls, Dys^WT^, mice were inoculated by intraperitoneal injection with 0.5 x 10^5^ PFU of CVB3 at 5 to 6 weeks of age. They were sacrificed on day 8 post-infection.

### Transthoracic Echocardiography

Mice (n = 21 for Dys^WT^ and n = 23 for Dys^KI^) were assessed for cardiac function measured by transthoracic echocardiography performed at 8 days post-infection. In order to accommodate a thorough examination, animals were anesthetized with 5% isoflurane for 30 seconds, then maintained at 0.5% throughout the examination. Small needle electrodes were placed into one upper and one lower limb to allow for a simultaneous electrocardiogram. The transthoracic echocardiography was performed using the FUJIFILM VisualSonics, Inc., Vevo 2100 ultrasound system with a 32-55MHz linear transducer. Heart rate (HR), left ventricular end-diastolic dimensions (LVED), left ventricular end-systolic dimensions (LVESD), interventricular septal thickness at end-diastole (IVSd), and LV posterior wall thickness at end-diastole (LVPWd) were determined through LV M-mode tracing. Percentage fractional shortening (%FS) was used as an indicator of systolic cardiac function.

### Von Kossa Stain

Histology was performed by the UC San Diego Histology Core. Hearts were fixed in 10% formalin, then embedded in paraffin and dried at 60°C. Tissues with chips of un-decalcified calcium were used as a control. Samples were de-paraffinized and hydrated, then placed in 5% silver nitrate and exposed to an ultraviolet lamp for 30–60 min. Then samples were washed with distilled water, fixed in 2% HYPO (2g of sodium thiosulfate in 100mL distilled water) for 5 min, and washed again in distilled water. A counterstain was performed with 1% Neutral Red for 1 min and samples were quickly rinsed and dehydrated, then mounted. Calcium appears in the black to brown spectrum and nuclei appear red.

### Hematoxylin and Eosin, Picrosirius Red, and Prussian Blue Staining

Histology was performed by the UC San Diego Histology Core. Hearts were fixed in 10% formalin, then embedded in paraffin and dried at 60°C. Samples were sectioned and stained with respective agents.

### Quantitation of Extent of Myocardial Echogenicity

Extent of intramyocardial echogenicity was assessed in a blinded and independent manner by two observers with experience in echocardiography. The parasternal long axis view was used to allow for a more comprehensive view of the left ventricle. Extent of myocardial echogenicity was assessed and scored in a semi-quantitative manner using a scale of 0–4 (4 representing the highest reflectance with the greatest distribution throughout the ventricle and 0 representing normal myocardium).

### Micro-Computed Tomography (MicroCT) Image Analysis

Scanning and measurements were performed by Numira Inc, now ScanCo Medical. Samples were scanned on a high-resolution, volumetric microCT scanner (μCT40, ScanCo Medical, Zurich, CH). Each sample was scanned twice, once for acquiring calcium deposit data and once for soft tissue structural data with the following parameters: 6 μm isotropic voxel resolution at 300 ms exposure time, 2000 views, and 2 frames per view. After the first scan, samples were stained using a proprietary contrast agent for contrast-enhanced imaging of the soft tissue. MicroCT-generated DICOM (Digital Imaging and Communications in Medicine) files were used to analyze the samples and create volume renderings of the regions of interest. Raw data files were viewed using AltaViewer (Numira Inc.) and volume rendering images for each sample were generated with SCIRun (Scientific Computing and Imaging Institute, University of Utah). The raw data files were then converted into a file format compatible with the segmentation software VHLab (Numira, Salt Lake City, UT). Areas of calcium deposits were segmented out for each sample and SCIRun was used to register the soft tissue data to the calcium deposit data. Teem (http://teem.sourceforge.net/) was used to convert the label map and imaging data into frames for the planar as well as segmentation overlay movies. These movie frames were then converted into QuickTime (Apple Inc.) movies for viewing. After the segmentation process, volume measurements were obtained by calculating the voxel count associated with the region of interest using VHLab, then multiplying by the cubic voxel resolution.

### Statistics

Statistics were performed using GraphPad Prism 5.00 (GraphPad Inc., San Diego, CA). The significance of extent of echogenicity measurements was assessed using an unpaired T-test and Welch’s correction. Regression analysis was performed using correlation analysis in Prism and a p-value less than 0.05 was considered significant.

### Study Approval

All procedures were performed in accordance with guidelines established by the University of California San Diego Institutional Animal Care Program (ACP) and were approved by the University of California San Diego Institutional Animal Care and Use Committee (IACUC).

## Results

### Areas of Increased Echogenicity in CVB3 Infected Mice

**Fig 1:**
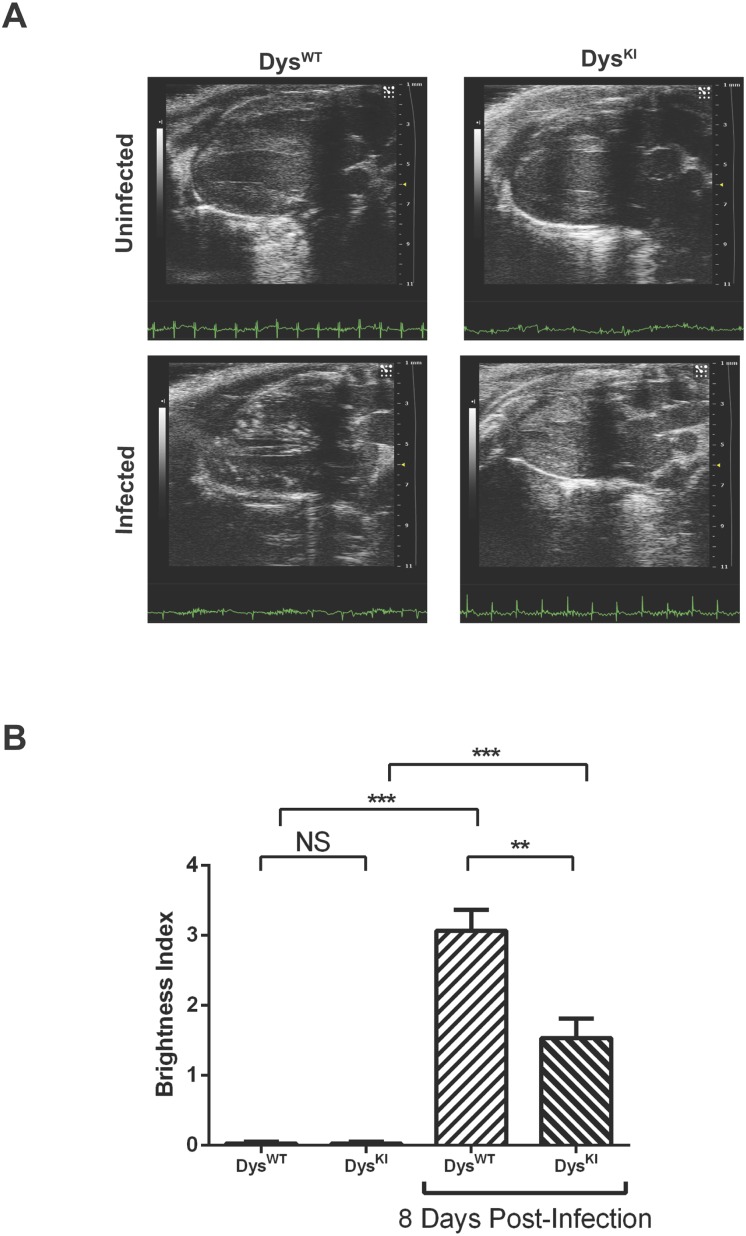
Increased echogenic foci in CVB3 infected mice. **(A)** Two-dimensional parasternal long axis echocardiograms in infected and uninfected mice. In uninfected mice (top panels), the left ventricular myocardium has a relatively homogenous echodensity. However, in infected Dys^WT^ mice (lower left panel) there are foci of increased echogenicity scattered throughout the ventricular myocardium. In infected mice in which the dystrophin-gene is mutated to prevent cleavage by CVB3 protease 2A, (lower right panel) the echogenic areas were less than in the infected WT-mice. (S1–S4 Videos are from the same echocardiograms.) **(B)** Using semi-quantitative grading of the echogenic areas (Echogenicity scored from 0–4) there was a significant increase in the infected hearts when compared to the uninfected hearts. Furthermore, the extent of echogenicity was lower in infected Dys^KI^ mice than the infected Dys^WT^ mice (N = 43 total mice, data is mean+/- s.e. ** p<0.01 ***p<0.001).

While performing echocardiography on CVB3 infected mice we observed punctate foci of increased echogenicity in the Dys^WT^, CVB3 infected mice (Fig 1A, left and S1–S4 Videos). The extent of the foci of increased echogenicity was assessed in a blinded, semi-quantitative manner on a scale of 0–4 by two individuals with experience evaluating mouse echocardiograms (Fig 1B).

We then sought to assess the echogenic foci in mice that were less susceptible to CVB3 infection. Coxackieviruses express a protease 2A, which is responsible for cleavage of the viral polyprotein and can act in trans on a small subset of host cellular proteins including dystrophin [10]. Dystrophin, a member of the Dystrophin-Glycoprotein Complex (DGC), links the sarcolemma and extracellular matrix with the cytoskeleton and is critical for maintaining sarcolemmal integrity. The cleavage of dystrophin is associated with disruption of the sarcolemmal membrane [11]. Mutation of the protease 2A-dystrophin cleavage site using knock-in of a mutation, Dys^KI^, prevents cleavage of dystrophin, decreases viral replication and decreases sarcolemmal disruption as demonstrated by a decrease in virus titer, inflammation, fibrosis and von Kossa staining [12]. Consistent with this phenotype, there were fewer foci of echogenicity in the Dys^KI^ mice when compared to the Dys^WT^ mice, even though the Dys^KI^ infected mice had significantly more foci of echogenicity than were observed in the uninfected Dys^KI^ mice, (Fig 1A and 1B). These data indicate that the increases in echogenicity were caused by the viral myocarditis and that there is less echogenicity when the viral infection is attenuated as noted in the Dys^KI^ mice. Even though the echogenic myocardial foci were readily apparent in CVB3 infected, there were no significant functional abnormalities in the same hearts as detected by routine 2-dimensional echocardiography (Table 1), thus demonstrating that the echogenic myocardial foci can be detected in the absence of echocardiographically detectable ventricular dysfunction.

**Table 1:** Echocardiographic functional analysis of the left ventricle. Data shown from mice 8 days post-infection. Values in both infected (Dys^WT^ n = 12, Dys^KI^ n = 14) and uninfected (Dys^WT^ n = 9, Dys^KI^ n = 9) mice are considered within a normal functional range. There were no statistically significant differences between groups. Data shown as mean ± SD.

Parameter	Dys^WT^ Uninfected	Dys^KI^ Uninfected	Dys^WT^ Infected	Dys^KI^ Infected
LVIDd (mm)	3.34 ± 0.28	3.10 ± 0.18	3.17 ± 0.25	3.11 ± 0.41
LVIDs (mm)	2.07 ± 0.33	1.83 ± 0.34	1.90 ± 0.30	1.83 ± 0.36
IVSd (mm)	0.61 ± 0.02	0.61 ± 0.04	0.65 ± 0.10	0.62 ± 0.06
LVPWd (mm)	0.60 ± 0.05	0.62 ± 0.05	0.64 ± 0.07	0.61 ± 0.08
%FS	38.4 ± 7.2	41.0 ± 8.9	40.1 ± 7.4	41.4 ± 6.9

LVIDd, left ventricular end-diastolic dimension; LVIDs, left ventricular end-systolic dimension; IVSd, interventricular septal thickness at end-diastole; LVPWd, left ventricular posterior wall thickness at end-diastole; %FS, percent fractional shortening.

### Pathological Assessment of Extent of Myocarditis

**Fig 2:**
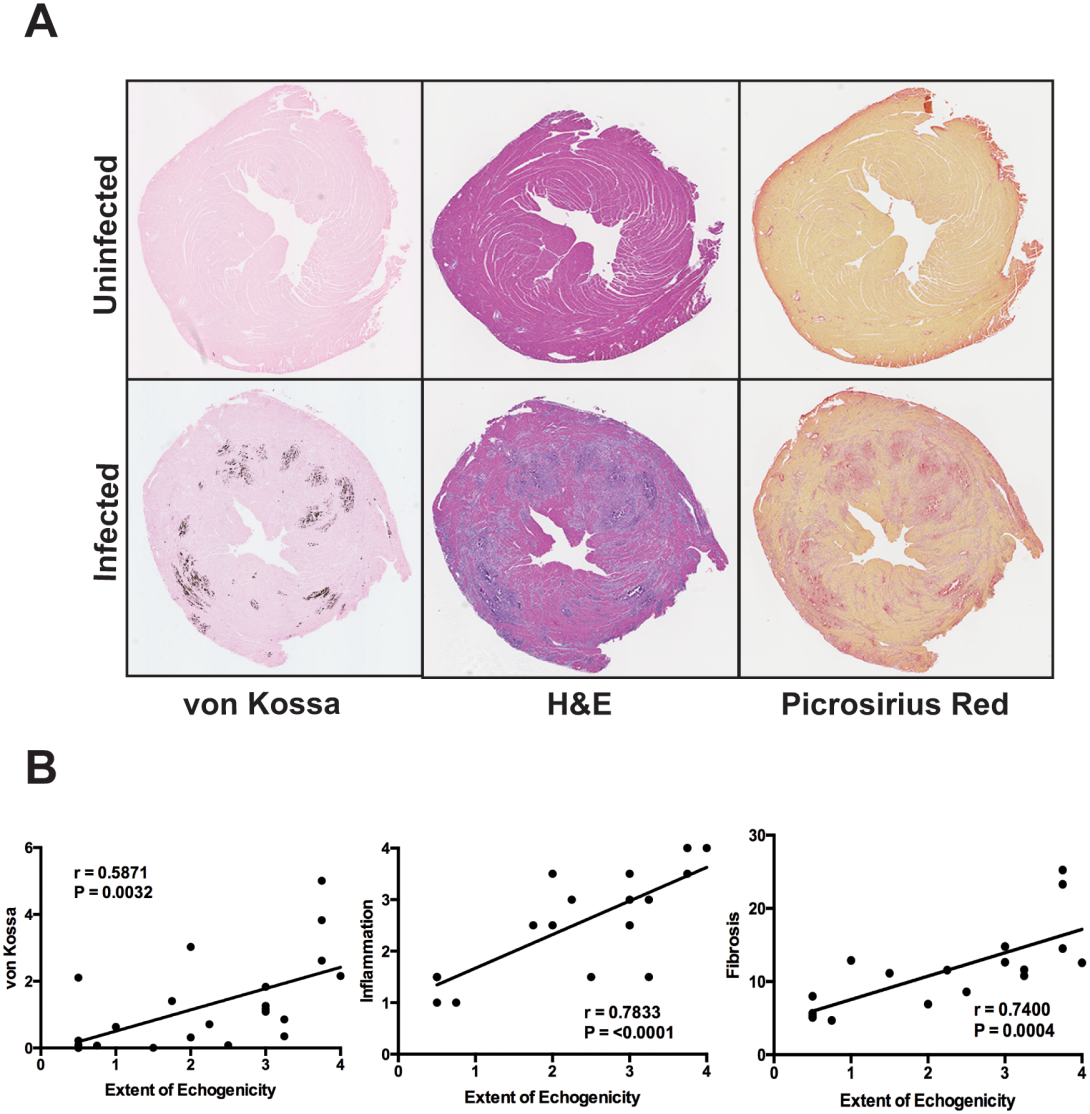
Correlation of extent of echogenicity with histologic markers of myocarditis. **(A)** von Kossa (left panels), hematoxylin and eosin (center panels), and picrosirius red (right panels) stains are shown from a representative uninfected and infected wild type mouse heart. In each case, abnormal staining was only present in infected mouse hearts. **(B)** The extent of von Kossa staining was quantitated as a percent of the total myocardial area that was von Kossa positive. The inflammation score was determined using a semi-quantitative scoring system (0–4) as previously described (12). Extent of fibrosis, as determined by picrosirius red staining, is expressed as a percent of total myocardial area. As shown in panel B, there was a significant correlation between extent of myocardial echogenicity and area of von Kossa staining, inflammation score, and fibrosis.

The extent of myocarditis was assessed using hematoxylin and eosin staining for inflammation, picrosirius red for fibrosis and von Kossa staining for myocardial calcium phosphate [13, 14]. Von Kossa staining identifies myocytes that have high levels of calcium phosphate [15]. It is generally thought to be a marker of myocardial cells undergoing necrosis with disruption of the sarcolemmal membrane. Similar to the increased echogenicity, there was a marked increase in von Kossa staining in infected Dys^WT^ mice that underwent echocardiographic analysis. All three markers of myocarditis, inflammation, fibrosis and von Kossa stain, correlated with the extent of echocardiographically determined myocardial echogenicity (Fig 2). In addition, the extent of von Kossa staining in infected Dys^KI^ mice was significantly less than that observed for the Dys^WT^ mice (data not shown) as previously demonstrated [12]. The correlation between echogenicity and the three pathologic markers of myocarditis indicate that the extent of echogenicity observed correlates with the overall severity of myocarditis. While this correlation is clear at 8 days post infection, it is possible that findings may be different at different time points through the course of viral myocarditis.

### Increased Radiodense Foci on MicroCT in CVB3 Infected Mice

**Fig 3:**
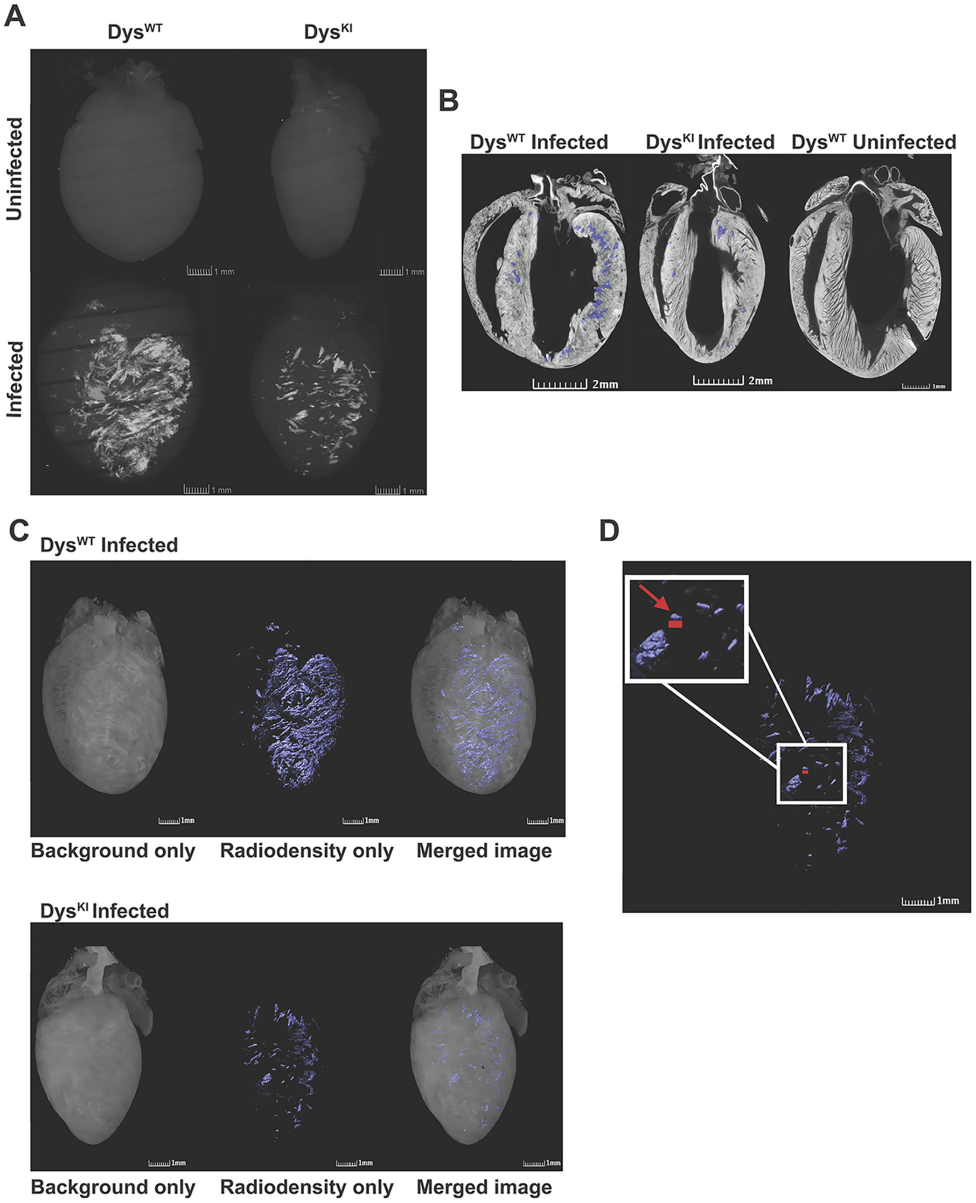
Increased radiodensity by microCT in CVB3 infected mouse hearts. **(A)** MicroCT images obtained from uninfected Dys^WT^ and Dys^KI^ are shown in the upper panels. MicroCT images from infected Dys^WT^ and Dys^KI^ are in the lower panels. The white areas are the areas of increased radiodensity. **(B)** Tomographic images of hearts from infected Dys^WT^ and Dys^KI^ and an uninfected Dys^WT^ mice were visualized with a background stain that is visible on microCT (white) to identify the cardiac structures. The areas of radiodensity are superimposed in blue in these images. **(C)** MicroCT images localize the radiodense areas in the heart of Dys^WT^ (top) and Dys^KI^ (bottom) infected hearts. Background staining alone is shown in the left images. The radiodense foci are shown in the middle images (blue), and the merged images are shown on the right. **(D)** Zoomed image of the three-dimensional reconstruction of the microCT of the infected Dys^KI^ heart from the middle of the lower panel in C demonstrates that areas of radiodensity can be detected when as small as the 100 x 40μm as represented by the red bar (arrow). 1 mm scale bars are shown in the bottom right of each image.

With the observation of echogenic regions and positive von Kossa staining in CVB3 infected mice, we hypothesized that microCT imaging analysis would allow for precise 3-D localization of foci of calcification. Accordingly, *ex vivo* whole hearts underwent microCT image analysis (Numira, Inc). High resolution radiodense foci were present in infected Dys^WT^ and Dys^KI^ mouse hearts (Fig 3A, bottom). There were minimal to no radiodense foci in the uninfected Dys^WT^ and Dys^KI^ mouse hearts (Fig 3A, top. See rotating 3-D images, S5–S8 Videos). We acknowledge that there is a limitation in distinguishing between calcium and iron on microCT, so we performed Prussian Blue staining and found no significant evidence of iron deposition in this model of myocarditis (S1 Fig). To more precisely define the location of the radiodense foci in the myocardium the myocardial tissue was counterstained. Radiodense foci were clearly localized to the ventricular myocardial wall as shown in Dys^WT^ and Dys^KI^ infected mice as compared with an uninfected mouse (Fig 3B, radiodense foci shown as blue, background stain is white. S9–S11 Videos). Three dimensional reconstruction of the microCT images demonstrates that the radiodensities associated with viral infection can be localized in a pattern that is consistent with myocardial fiber orientation. The width of the fiber-like structures is often as little as 40 μm (arrow). This is similar to the width of an individual myocyte. (Fig 3C and 3D and S12 and S13 Videos). In a manner similar to that observed in the echocardiograms and von Kossa staining, the microCT image analysis demonstrates that the foci of increased radiodensity are scattered throughout the ventricular myocardium. The volume of myocardial radiodensities can be precisely quantified using microCT images (Table 2).

**Table 2:** Volume measurements of radiodense foci. Table demonstrates the volume of the heart tissue compared to the volume of radiodense foci and the percent of the volume of the heart tissue that contains radiodense foci in the infected and uninfected Dys^WT^ and Dys^KI^ hearts shown in the figures. Each measurement is from a single heart.

Sample	Type	Radiodense Foci Volume (mm^3^)	Heart Tissue Volume (mm^3^)	RDV/HV (%)
796	Dys^WT^ uninfected	0.000	84.59	0.00
795	Dys^KI^ uninfected	0.000	67.28	0.00
808	Dys^WT^ infected	4.019	106.29	3.78
805	Dys^KI^ infected	0.521	73.44	0.71

RDV/HV, radiodense foci volume/ heart tissue volume.

## Discussion

The results of these experiments demonstrate that novel imaging approaches can be used to assess quantitatively the degree of myocardial injury in murine myocarditis. Following CVB3 infection in the mouse we clearly demonstrate an increase in echogenic foci within the myocardium that occur in the absence of echocardiographically detectable abnormalities in ventricular function. These foci do not occur in uninfected mice. Consistent with the observation that Dys^KI^ mice are more resistant to CVB3 infection [12] when compared to Dys^WT^ mice, CVB3 infected Dys^KI^ mice show significantly fewer echogenic foci than their infected Dys^WT^ littermates. The extent of echogenic foci within the myocardium correlates with the extent of inflammation, fibrosis and von Kossa staining based on analysis of the CVB3 infected Dys^WT^ and Dys^KI^ groups. Furthermore, we demonstrate, for the first time, that CVB3 infection can be imaged in 3-dimensions using microCT at a resolution as low as 40 μm, a size that approximates that of a single myocardial cell. While it was not possible to precisely co-localize the von Kossa stain with the radiodense areas in the myocardium due to technical limitations, it is highly likely that the radiodense areas represent myocardial cells that have taken up calcium, a marker of myocardial cell necrosis [15]. Iron staining has been demonstrated in some forms of myocarditis [16], but we did not observe uptake of iron in our experiments (S1 Fig). By utilizing microCT, we were able to precisely assess the volume of myocardial calcium uptake and accurately locate affected myocytes. It was interesting to note that the pattern of radiodensity and presumably myocardial infection, appears to be along the myofibers. This is consistent with infection propagating through the intercalated discs where high concentrations of the viral receptor, CAR are known to be located [17].

Myocarditis is decreased in the heart when CVB3 is not able to cleave Dystrophin as demonstrated in the CVB3 infected Dys^KI^ mouse heart [13]. In this genetically modified model, a point mutation was “knocked-in” to the protease 2A cleavage site, thus preventing protease 2A-mediated cleavage of Dystrophin. As had been previously demonstrated using histologic techniques, in the current study we were able to demonstrate a decrease in the extent of myocarditis using a semi-quantitative assessment of the extent of echogenic foci that were present in the ventricular myocardium. The prevalence of the echogenic foci in infected Dys^WT^ and Dys^KI^, correlate by regression analysis with the extent of myocarditis in the same mice as assessed by inflammation, fibrosis, and von Kossa histological staining. In regards to specificity of these findings, we have occasionally observed areas of echogenicity in models of myocardial infarction or ischemia/reperfusion with patterns that are different than observed in myocarditis. We have not observed similar findings in other models of cardiomyopathy such as pressure overload (transverse aortic constriction) or genetically modified models of cardiomyopathy.

By focusing on calcium accumulation within damaged myocytes, we were able to use microCT analysis as a novel method for assessing calcification in the CVB3-infected heart. MicroCT analysis revealed apparent foci of calcium in heart samples with 3.78 and 0.71% of the myocardium showing calcification in an infected wild-type mouse and knock-in mice, respectively, compared to 0% in the uninfected controls (Table 2). These positive microCT results provide insight into myocardial calcification that can occur during CVB3 infection. This technique may also be a useful diagnostic tool in other diseases, such as Duchenne Muscular Dystrophy, that are characterized by cell membrane deficiency and Ca^2+^ mishandling.

Previous work related to tissue characterization in humans has indicated that digital image texture analysis of echocardiograms may be useful in distinguishing between myocarditis and normal myocardium [18–20]. However, as the use of MRI has improved the diagnosis of myocarditis in humans, echocardiographic tissue characterization of the myocardium in patients has not been broadly pursued [21]. There are instances in the clinic where myocardial calcification has been observed [16, 22–24]. The etiology can vary from infectious or immune-mediated myocarditis to myocardial infarction [25]. While there is moderate evidence that CT detection of certain cases of myocarditis is possible in a clinical setting, the diagnostic accuracy has not been fully assessed. The images presented in the current study demonstrate that microCT imaging yields very high resolution images, which correlate with the level of myocarditis in our animal model. However, the applicability of microCT imaging to a clinical setting is limited by the *ex vivo* nature of the microCT technique and the ability of microCT to image voxels that are much smaller than traditional CT imaging. As the resolution of CT imaging improves, it may be possible to more clearly identify clinical myocarditis associated with myocardial calcification.

## Supporting Information

S1 Fig Prussian Blue Staining. Prussian Blue staining for infected and uninfected samples. The positive control is an uninfected spleen.
(TIF)

S1 Video Echocardiography. Parasternal long axis echocardiography of uninfected Dys^WT^ mouse.
(MOV)

S2 Video Echocardiography. Parasternal long axis echocardiography of uninfected DysKI mouse.
(MOV)

S3 Video Echocardiography. Parasternal long axis echocardiography of CVB3 infected Dys^WT^ mouse.
(MOV)

S4 Video Echocardiography. Parasternal long axis echocardiography of CVB3 infected Dys^KI^ mouse.
(MOV)

S5 Video microCT. 3D, rotating microCT reconstruction of uninfected Dys^WT^ mouse.
(MOV)

S6 Video microCT. 3D, rotating microCT reconstruction of uninfected Dys^KI^ mouse.
(MOV)

S7 Video microCT. 3D, rotating microCT reconstruction of CVB3 infected Dys^WT^ mouse.
(MOV)

S8 Video microCT. 3D, rotating microCT reconstruction of CVB3 infected Dys^KI^ mouse.
(MOV)

S9 Video microCT. Coronal cross-section of counterstained CVB3 infected Dys^WT^ mouse.
(MOV)

S10 Video microCT. Coronal cross-section of counterstained CVB3 infected Dys^KI^ mouse.
(MOV)

S11 Video microCT. Coronal cross-section of counterstained uninfected Dys^WT^ mouse.
(MOV)

S12 Video microCT. 3D, counterstained, rotating microCT reconstruction of CVB3 infected Dys^WT^ mouse.
(MOV)

S13 Video microCT. 3D, counterstained, rotating microCT reconstruction of CVB3 infected Dys^KI^ mouse.
(MOV)
