# Supplementary figures and images for: Increased Echogenicity and Radiodense Foci on Echocardiogram and MicroCT in Murine Myocarditis
Source: PLoS One. 2016 Aug 3;11(8):e0159971. doi: 10.1371/journal.pone.0159971 (PMC4972301; doi:10.1371/journal.pone.0159971)

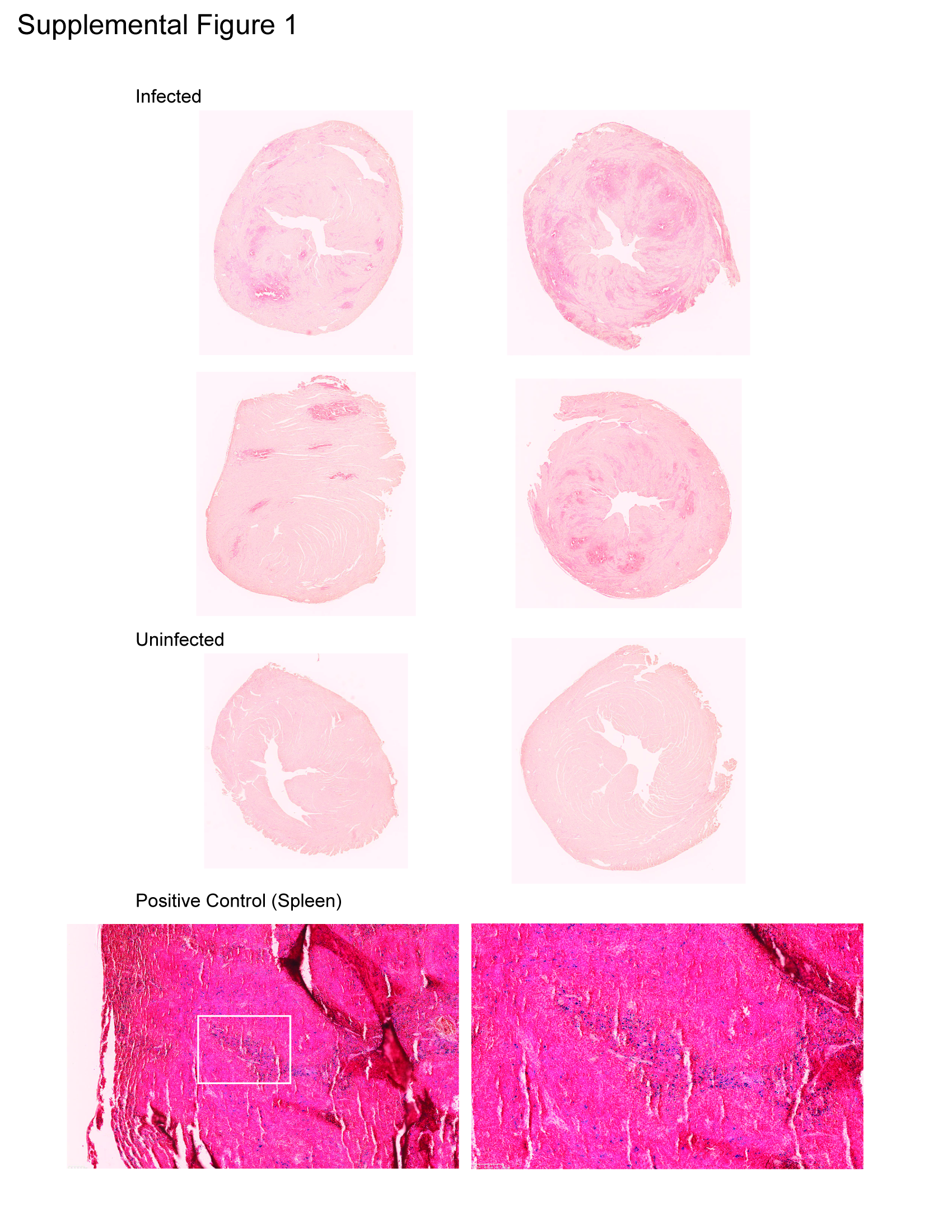

Supplement: S1 Fig — Prussian Blue staining for infected and uninfected samples. The positive control is an uninfected spleen. (TIF) [file pone.0159971.s001.tif]
